# Effect of increasing levels of threonine relative to lysine on the performance and meat quality of finishing pigs

**DOI:** 10.5713/ab.21.0078

**Published:** 2021-06-23

**Authors:** Santi Devi Upadhaya, Sang Seon Lee, Sung Giu Jin, Zhenlong Wu, In Ho Kim

**Affiliations:** 1 Department of Animal Resource and Science, Dankook University, Cheonan 31116, Korea; 2 Department of Pharmaceutical Engineering, Dankook University, Cheonan 31116, Korea; 3 State Key Laboratory of Animal Nutrition, Department of Animal Nutrition and Feed Science, China Agricultural University, Beijing 100193, China

**Keywords:** Finishing Pig, Lysine, Meat Quality, Performance, Threonine Ratio

## Abstract

**Objective:**

The present study aimed to evaluate the effects of varying standardized ileal digestible lysine:threonine (SID Lys:Thr) ratio in the diet on the performance and meat quality of finishing pigs.

**Methods:**

In total 192 crossbred pigs ([Landrace×Yorkshire]×Duroc, 17 weeks old), with an initial body weight (BW) of 70.6±3.9 kg were used in an 8-wk trial. Pigs were randomly allotted to one of six dietary treatments based on their initial BW and sex (8 replications; 4 pigs per pen, 2 barrows and 2 gilts). The pigs in the 6 treatments were fed diets having different SID Lys:Thr ratios such as 1:0.65, 1:0.66, 1:0.67, 1:0.68, 1:0.69, and 1:0.70.

**Results:**

A linear increment (p<0.05) in average daily gain (ADG) and trends in reduction in feed conversion ratio (FCR) were observed during day 29 to 56 of the experiment and the apparent total tract digestibility (ATTD) of dry matter tended to increase linearly (p = 0.094) at the end of the experiment (day 56) with the increase in the dietary SID Lys:Thr ratios. The backfat thickness and lean percentage increased (linear effect, p<0.05) on day 28. In addition, at day 56, a linear (p<0.05) increment in lean percentage was observed. Significant quadratic responses (p = 0.02) for pH and drip loss at day 7 (p = 0.02), a linear increase (p<0.05) in cooking loss and drip loss at day 7, and a trend in quadratic response (p = 0.07) in the lightness of meat color (L*) were observed, whereas other meat quality indices were unaffected by varying the SID Lys:Thr ratios.

**Conclusion:**

The SID Lys:Thr ratio for maximum ADG, minimum FCR and enhanced digestibility was found to be 0.70. However, for carcass trait and meat quality, the SID Lys:Thr ratio of 0.65 was enough.

## INTRODUCTION

Threonine is an essential amino acid (AA) that is not synthesized by animals and is required for both maintenance and growth. It is the primary AA constituent of immunoglobulins and represents the proteins that are secreted in a significant proportion by the small intestine [1]. A sufficient intake of dietary threonine (Thr) has been reported to be particularly important for maintaining the integrity of the gut barrier because of its role in the synthesis of mucosal mucin in pigs [2]. Threonine is also an important AA for protein synthesis, and its catabolism generates important metabolites such as glycine, acetyl-CoA, and pyruvate [3]. The ratio for some AAs relative to lysine (Lys) is assumed to increase with increasing body weight (BW), because of varying requirements for maintenance and production. For instance, the Thr requirement for maintenance has been suggested to be relatively high and varies with the age, rate of growth or protein accretion [4,5]. The ileal digestibility of AAs among different feedstuffs varies widely [6,7]. The possible reason for the variation in Thr requirement may be that some of the older studies were based on the concentration of total Thr in the diet rather than the ileal digestible Thr concentration [8,9]. By using AA ideal protein ratios, it is assumed that the amount of AAs provided to animals is enough to meet the requirements for maintenance and growth without creating a surplus or deficiency [10]. For reducing nitrogen (N) excretion and feed costs, the concept of utilizing ideal protein is widely accepted as an effective tool. A previous study suggested that the ideal AA ratios for Thr range from 65% of Lys for 5 to 20 kg live weight to 70% of Lys requirement for 50 to 100 kg live weight [11]. This implies different AA requirements for different BWs. In another study by Plitzner et al [12], the total dietary Lys:Thr ratio was estimated to be 1:0.65 or higher for modern crossbred finishing pigs (68 to 113 kg) based on the concept of ideal protein. The results of published studies give a wide range for Thr requirements of pigs in all stages of growth.

The present study aimed to evaluate the effects of increasing the level of Thr by 1% unit from 65% of Lys requirements in CON/basal diet to 5 different levels (66%, 67%, 68%, 69%, and 70% of Lys) to evaluate the optimal SID Lys:Thr ratio which enhances the performance and carcass quality of finishing pigs

## MATERIALS AND METHODS

### Animal care

This study was undertaken following the guidelines of the Research Policy of Dankook University. The protocol for this study (DK-2-2004) was approved by the Animal Care and Use Committees of Dankook University, Cheonan, South, Korea.

### Animals and diets

A total of 192 crossbred pigs ([Landrace×Yorkshire]×Duroc, 17 weeks old) with an initial BW of 70.6±3.9 kg were used in an 8-wk trial. Pigs were randomly allotted to one of six dietary treatments (each n = 32) based on their initial BW and sex (8 replications; 4 pigs per pen, 2 barrows and 2 gilts). The pigs in the 6 treatments were fed with diets having different Lys:Thr ratios: 1:0.65, 1:0.66, 1:0.67, 1:0.68, 1:0.69, and 1:0.70 by supplementing the diet with increasing levels of synthetic Thr. Threonine used in the present study was purchased from NL FARM, 318, RIT center, Gyeonggi Technopark, Ansan-si, Gyeonggi-do, Republic of Korea.

The diets were formulated to be isoenergetic and isonitrogenous and to meet or exceed the NRC [13] nutrient requirements (Table 1). All pigs were housed in an environmentally controlled room with a slatted plastic floor. Each pen was equipped with a 1-sided self-feeder and a nipple waterer to allow the pigs *ad libitum* access to feed and water throughout the experimental period.

### Growth performance

Feed intake per pen was recorded daily, individual pig BW was measured on the initial day, at the end of week 4 (day 28) and week 8 (day 56) of the experiment, the feed consumption, and pig BW were used to monitor ADG, average daily feed intake (ADFI) and feed conversion ratio (FCR).

### Nutrient digestibility

The apparent total tract digestibility (ATTD) of nutrients was measured at the end of the experimental period. Chromium oxide (Cr_2_O_3_, 0.25%), an indigestible marker was added to the diet 7 days prior to fecal collection to calculate the nutrient digestibility. Fecal samples were randomly collected from at least two pigs/pen (n = 16 per treatment) via the rectal massage and were pooled by pen. Based on treatment, all the feed and fecal samples were dried (70°C for 72 h) and finely ground to be able to pass through a 1 mm screen. Then fecal samples were analyzed for dry matter (DM) and N following the procedure outlined by AOAC International [14]. The gross energy was determined by measuring the heat of combustion by Parr 6400 oxygen bomb calorimeter (Parr Instrument Co., Moline, IL, USA). Chromium was analyzed via UV/VIS spectrophotometer (Optizen POP, Daejeon, Korea). For calculating the ATTD of the nutrients, we used the following formula: Digestibility = 1–([Nf×Cd]/[Nd×Cf]) ×100, where Nf = concentration of nutrient in feces (% DM), Nd = concentration of nutrient in the diet, Cd = concentration of chromium in the diet and Cf = concentration of chromium in the feces.

Feed samples were analyzed for crude protein (N×6.25; method 988.05), crude fat (method 954.02), crude fiber (method 962.09), crude ash (method 942.05), calcium (method 984.01), phosphorous (method 965.17), and AAs (method 982.30E) following the procedures established by AOAC International [14].

### Backfat thickness and lean percentage

The back fat thickness and lean percentage of all live pigs (n = 32 per treatment) were measured 5 cm from the right-hand side of the midline from 3 different sites (shoulder, mid back, and loin at a position directly above the point of elbow, last rib, and last lumbar vertebra, respectively) before the commencement and at the end of week 4 and week 8 of the experiment using a real-time ultrasound instrument (Piglog 105; SFK Technology, Herlev, Denmark [15]). The mean value was taken and used for subsequent statistical analysis.

### Meat quality

At the end of the experiment, animals were transported to a local abattoir and slaughtered. After slaughter, carcass weight and backfat thickness were measured from all pigs. Ten pigs per treatment (total 60 pigs) were randomly selected prior to the transport to the abattoir for meat quality assessment. After slaughter, carcasses from the selected pigs were placed in a conventional chiller at 4°C. Then carcasses were fabricated into primal cuts after a 24-h chilling period. Meat samples including lean, and fat were taken via perpendicular cut loins into 2-cm-thick chop beginning from the 10th and 11th ribs region and transported to the laboratory. The pH of muscle was measured 24 h after postmortem using a pH meter (Testo 205, Testo, Lenzkirch, Germany). Sensory evaluation was conducted by trained panelists to evaluate the color darkness, firmness and marbling of fresh loin samples using a five-point assessment scheme according to the procedures established by the National Pork Producers Council (NPPC) [16]. Immediately after the subjective tests were conducted, meat color of the longissimus muscle (LM) as lightness (L*), redness (a*), and yellowness (b*), was determined using a Minolta Chroma Meter (CR-210, Minolta, Tokyo, Japan) to evaluate the freshly cut surface after 30 min of blooming at 4°C. Water-holding capacity (WHC) was measured using methods of Kauffman et al [17]. Briefly, a 0.2-g sample was pressed at 3,000 psi for 3 min onto laboratory grade125-mm-diameter filter paper. The areas of pressed sample and expressed moisture were delineated and determined with a digitizing area-line sensor (MT-10S; M. T. Precision Co. Ltd, 123 Tokyo, Japan). A ratio of water area: meat area was calculated to measure WHC, with smaller ratio indicating higher WHC. The LM area was measured by tracing the LM surface at the 10th rib, which also used the mentioned above digitizing area-line sensor. Cook loss was determined as described previously by Sullivan et al [18]. Briefly, 5 g of meat sample were heat-treated in plastic bags separately in a water bath (100°C) for 5 min. Samples were cooled at room temperature. Cooking loss was calculated as (sample weight before cooking - sample weight after cooking)/sample weight before cooking×100. According to the plastic bag method described by Honikel [19], drip loss was measured using ~4 g of meat sample.

### Statistical analysis

Data were analyzed as a completely randomized-block design using the general linear model procedures (SAS Institute Inc., Cary, NC, USA). The pen served as the experimental unit for all response criteria except meat quality wherein individual pigs were the experimental unit. Variability in data is expressed as standard errors of the mean. Linear and quadratic polynomial contrasts were performed to determine the effects of the different SID Lys:Thr ratios in the diet, with p<0.05 indicating significance and p<0.10 indicating trends.

## RESULTS

### Growth performance

Increasing the SID Lys:Thr ratio of finishing diets did not exert any significant effects in ADG, ADFI, and FCR during day 1 to 28 and in the overall experiment period although numerical increase in ADG and a slight reduction in FCR was observed during these periods. However, increasing the SID Lys:Thr ratio of finishing diets led to a numerical increase in BW during the end of the experiment. In addition, a significant linear increment in ADG (p<0.05) and trends in reduction in FCR but no effect on ADFI was observed during day 29 to 56 (Table 2).

### Apparent total-tract nutrient digestibility

The ATTD of DM tended to increase linearly (p = 0.094) at the end of the experiment (day 56) with the increase in the SID Lys:Thr ratio of finishing diets. The ATTD of N and energy remained unaffected with the increase in SID Lys:Thr ratio although there were numerical increments in digestibility values (Table 3).

### Backfat and lean percentage

The backfat thickness and lean percentage increased significantly (linear effect, p<0.05) with the increase in the SID Lys:Thr ratio of finishing diets on day 28. In addition, at day 56, a trend in a linear increase in backfat thickness (p = 0.073) and a significant linear (p<0.05) increment in lean percentage was observed as the ratio of SID Lys:Thr increased in the diet of finishing pigs (Table 4).

### Carcass trait and meat quality

There were no significant effects on carcass weight and backfat thickness of pigs at slaughter by increasing the SID Lys:Thr ratio in finishing diet. Significant quadratic responses (p = 0.02) for pH and drip loss at day 7 (p = 0.02), a significant linear increase (p<0.05) in cooking loss and drip loss at day 7, and a trend in quadratic response (p = 0.07) in the lightness of meat color (L*) were observed, whereas other meat quality indices were unaffected by varying SID Lys:Thr ratio in finishing diet (Table 5).

## DISCUSSION

The objective of the present study was to determine the effects of increasing dietary SID Thr:Lys ratio into isoenergetic and isonitrogenous finishing pig diets on their performance and carcass quality. Several previous studies have reported the varying optimal Lys:Thr ratios. For instance, a study by Xie et al [20] using a linear-plateau model showed that the optimum SID Thr:Lys ratios for maximal weight gain and minimal feed conversion were 67% and 71%, respectively in finishing pigs weighing 72 kg at the start of the experiment when fed low crude protein diets *ad libitum*. In another study, Ma et al [21] reported that 90 to 118 kg gilts when fed low crude protein diets *ad libitum*, the optimum SID Thr:Lys ratios to maximize average daily gain and to minimize FCR were 61% and 63%, respectively, using a linear-plateau model. Based on recent literature, van der Peet-Schwering and Bikker [22] recommended a minimum SID Thr:Lys ratio from 66% to 68% for growing and finishing pigs which agrees with the findings of Plitzner et al [23]. Results from the present study have shown that dietary Thr concentration of 7.0 g/kg improved daily gains by 5.9% and the FCR by about 3.6% points compared to pigs fed the basal diet (6.5 g/kg Thr) during day 29 to 56 which is the highest values among different Thr concentrations tested in this study. A cumulative improvement in ADG and FCR by about 3.7% and 2.4% points respectively was observed in pigs receiving dietary Thr concentration of 7.0 g/kg compared to those receiving basal diet (6.5 g/kg Thr). The SID Lys:Thr ratio for growth performance in the present study corroborates the findings of van Lunen [11]. The improvement in ADG and FCR in the present study the with the inclusion of 7.0 g/kg Thr in the diet might be due to the efficient absorption and utilization of other AAs at the given dose or it may be due to maintaining the adequate amount of mucosal mucins and protein synthesis [2].

Digestibility is an important factor in the measure of the nutrition value of animal feed. In the present study, increasing the SID Lys:Thr ratio in the finishing diet tended to linearly increase the DM digestibility and numerically increase in N and energy digestibility suggesting that 7.0 g/kg Thr is the best among the other tested concentrations. Since Thr plays an important role in mucin synthesis and barrier integrity maintenance [24,25], the inclusion of 7.0 g/kg Thr in the diet might have led to better villus surface and therefore better absorption and digestion. In addition, adequate Thr inclusion in the diet has been suggested to increase the abundance of potential beneficial bacteria which can eventually improve the nutrient digestion [26]. The trends in improvement in the digestibility of nutrients with the increase in the SID Lys:Thr ratio might have contributed in enhancing the performance of pigs.

As a pig grows towards its mature body size and weight, it deposits relatively more fat and less muscle. The backfat and lean percent measured in live pigs during week 4 and week 6 of the trial showed linear increments with the increase in the Lys:Thr ratios of finishing diet in the present study. However, Kobayashi et al [27] noted that the response to increasing level of Thr on the intramuscular fat in porcine muscle is negligible. Since there are no other reports of Lys:Thr ratios regarding their effects on backfat and lean percentage of live pigs, comparisons could not be made.

If the nutrient requirements of high genetic potential pigs are not met, it may cause them to have higher backfat at slaughter. Under appropriate feeding management and marketing time, a high-quality pork can be produced [28,29]. The influence of Thr supply on carcass characteristics has been reported to be much lower than the influence on growth performance [12]. In the present study, the carcass weight and backfat thickness at slaughter were unaffected by altering SID Lys:Thr ratios in the diet suggesting that 6.5 g/kg Thr was sufficient since the backfat values were the lowest at this concentration. Meat that exhibits low backfat and body fat, with excellent feed efficiency and meat productivity [30–32] is considered high quality. Leaner pig meat is associated with greater meat loss, lower fat, firmness, and less juiciness and flavor [28,32,33]. In addition, the linear response in pH, cook loss, and quadratic response in L* values in the present study also suggest that SID Lys:Thr ratio of 65% is appropriate for good meat quality. These observations on carcass and meat quality support the theory that the Thr requirement for growing pigs is substantially higher for maintenance than for protein accretion [4,34].

## CONCLUSION

Taken together from the present findings, we conclude that the dietary SID Thr requirement for both maximum average daily gain and minimum FCR was 70% of Lys requirement and an optimum SID Lys:Thr ratio was also 0.70 to maximize nutrient digestibility. However, for carcass trait and meat quality, the SID Lys:Thr ratio of 0.65 was enough. These ratios are higher than the current recommendation of 0.63 for 75 to 100 kg finishing pigs [13].

## Figures and Tables

**Table 1:** Ingredient and nutrient composition of experimental diets for finishing pigs (as fed-basis)

Items	Level of Thr (% of requirements)					

	65	66	67	68	69	70

	CON^1)^	Thr1^1)^	Thr2^1)^	Thr3^1)^	Thr4^1)^	Thr5^1)^
Ingredients (%)						
Corn	76.785	76.776	76.758	76.739	76.731	76.712
Soybean meal (48%)	15.71	15.71	15.71	15.72	15.72	15.72
Tallow	2.46	2.46	2.47	2.47	2.47	2.48
Molasses	2	2	2	2	2	2
Dicalcium phosphate	1.35	1.35	1.35	1.35	1.35	1.35
Limestone	0.56	0.56	0.56	0.56	0.56	0.56
Salt	0.3	0.3	0.3	0.3	0.3	0.3
Methionine (99%)	0.02	0.02	0.02	0.02	0.02	0.02
Lysine	0.29	0.29	0.29	0.29	0.29	0.29
Threonine (99%)	0.045	0.054	0.062	0.071	0.079	0.088
Mineral mix^2)^	0.2	0.2	0.2	0.2	0.2	0.2
Vitamin mix^3)^	0.2	0.2	0.2	0.2	0.2	0.2
Phytase	0.05	0.05	0.05	0.05	0.05	0.05
Choline (25%)	0.03	0.03	0.03	0.03	0.03	0.03
Total	100	100	100	100	100	100
Calculated composition (%)						
Metabolizable energy (kcal/kg)	3,300	3,300	3,300	3,300	3,300	3,300
Crude protein	14	14	14	14	14	14
Ca	0.6	0.6	0.6	0.6	0.6	0.6
P	0.55	0.55	0.55	0.55	0.55	0.55
Lys	0.86	0.86	0.86	0.86	0.86	0.86
Met	0.25	0.25	0.25	0.25	0.25	0.25
Thr	0.559	0.568	0.576	0.585	0.593	0.602
Trp	0.15	0.15	0.15	0.15	0.15	0.15
Crude fat	5.32	5.32	5.33	5.33	5.33	5.34
Crude fiber	2.35	2.35	2.35	2.35	2.35	2.35
Crude ash	4.36	4.36	4.36	4.36	4.36	4.36
Analyzed composition^4)^ (%)						
Crude protein	13.96	13.95	13.94	14.03	13.93	13.98
Ca	0.60	0.58	0.62	0.59	0.61	0.58
P	0.55	0.54	0.55	0.53	0.55	0.53
Lys	0.86	0.87	0.85	0.84	0.86	0.83
Met	0.22	0.24	0.25	0.25	0.24	0.23
Thr	0.560	0.567	0.578	0.585	0.593	0.601
Trp	0.15	0.16	0.14	0.14	0.15	0.15
Crude fat	5.32	5.31	5.33	5.32	5.34	5.34
Crude fiber	2.35	2.36	2.33	2.36	2.32	2.34
Crude ash	4.32	4.36	4.35	4.33	4.35	4.36

1) CON, lysine:threonine = 1:0.65; Thr1, lysine:threonine = 1:0.66; Thr 2, lysine:threonine = 1:0.67; Thr 3, lysine:threonine = 1:0.68; Thr 4, lysine:threonine = 1:0.69; Thr 5, lysine:threonine = 1:0.70.

2) Provided per kg diet: Fe, 115 mg as ferrous sulfate; Cu, 70 mg as copper sulfate; Mn, 20 mg as manganese oxide; Zn, 60 mg as zinc oxide; I, 0.5 mg as potassium iodide; and Se, 0.3 mg as sodium selenite.

3) Provided per kilograms of diet: vitamin A, 13,000 IU; vitamin D_3_, 1,700 IU; vitamin E, 60 IU; vitamin K_3_, 5 mg; vitamin B_1_, 4.2 mg; vitamin B_2_, 19 mg; vitamin B_6_, 6.7 mg; vitamin B_12_, 0.05 mg; biotin, 0.34 mg; folic acid, 2.1 mg; niacin, 55 mg; D-calcium pantothenate, 45 mg.

4) Data are the mean of duplicate analyses of each ingredient.

**Table 2:** Effect of varying dietary Lys:Thr ratios on growth performance in finishing pigs^1)^

Items	Level of Thr (% of Lys requirements)						SEM	p-value	

	65	66	67	68	69	70			
	
	CON^1)^	Thr1^1)^	Thr2^1)^	Thr3^1)^	Thr4^1)^	Thr5^1)^		Linear	Quadratic
Body weight (kg)									
Initial	70.54	70.57	70.56	70.56	70.56	70.57	0.04	0.724	0.989
Day 28	92.93	93.08	93.22	93.15	93.27	93.33	0.39	0.451	0.887
Day 56	115.71	116.04	116.31	116.39	116.63	117.45	0.79	0.119	0.746
Day 1 to 28									
ADG (g)	800.0	804.0	809.0	807.0	811.0	813.0	13.66	0.469	0.883
ADFI (g)	2,231	2,233	2,237	2,236	2,239	2,243	23.31	0.710	0.963
FCR	2.796	2.781	2.767	2.775	2.761	2.760	0.036	0.443	0.823
Day 29 to 56									
ADG (g)	814	820	825	830	834	862	14.55	0.023	0.448
ADFI (g)	2690	2694	2702	2708	2717	2744	35.02	0.246	0.698
FCR	3.309	3.288	3.280	3.268	3.260	3.190	0.045	0.075	0.561
Overall (Day 1 to 56)									
ADG (g)	807	812	817	818	823	837	14.04	0.122	0.746
ADFI (g)	2,461	2,464	2,469	2,472	2,478	2,494	25.06	0.323	0.77
FCR	3.055	3.038	3.026	3.025	3.015	2.981	0.035	0.145	0.813

Values represent the means of eight pens with four pigs per pen.

SEM, standard error of means; ADG, average daily gain; ADFI, average daily feed intake; FCR, feed conversion ratio.

1) CON, lysine:threonine = 1:0.65; Thr1, lysine:threonine = 1:0.66; Thr2, lysine:threonine = 1:0.67; Thr3, lysine:threonine = 1:0.68; Thr4, lysine:threonine = 1:0.69); Thr5, lysine:threonine = 1:0.70.

**Table 3:** Effect of varying dietary Lys:Thr ratios on nutrient digestibility in finishing pigs

Items (%)	Level of Thr (% of Lys requirements)						SEM	p-value	

	65	66	67	68	69	70			
	
	CON^1)^	Thr1^1)^	Thr2^1)^	Thr3^1)^	Thr4^1)^	Thr5^1)^		Linear	Quadratic
Day 56									
Dry matter	71.02	70.38	71.81	71.31	72.83	73.95	1.50	0.094	0.506
Nitrogen	69.56	69.06	70.26	70.00	70.72	71.40	1.84	0.373	0.814
Digestible energy	71.23	70.38	72.24	71.11	72.46	72.56	1.66	0.401	0.86

Values represent the means of eight pens with two pigs per pen.

SEM, standard error of means.

1) CON, lysine:threonine = 1:0.65; Thr1, lysine:threonine = 1:0.66; Thr2, lysine:threonine = 1:0.67; Thr3, lysine:threonine = 1:0.68; Thr4, lysine:threonine = 1:0.69); Thr5, lysine:threonine = 1:0.70.

**Table 4:** Effect of varying dietary Lys:Thr ratios on backfat thickness and lean percentage in finishing pigs

Items	Level of Thr (% of Lys requirements)						SEM	p-value	

	65	66	67	68	69	70			
	
	CON^1)^	Thr1^1)^	Thr2^1)^	Thr3^1)^	Thr4^1)^	Thr5^1)^		Linear	Quadratic
Initial									
Backfat thickness (mm)	12.05	11.94	11.96	11.81	11.99	12.03	0.12	0.916	0.212
Lean (%)	59.35	58.78	58.95	58.69	59.02	59.03	0.23	0.538	0.102
Week 4									
Backfat thickness (mm)	14.90	15.00	15.20	15.10	15.30	15.40	0.15	0.024	0.998
Lean (%)	55.68	55.80	55.96	55.94	56.08	56.17	0.19	0.044	0.881
Week 8									
Backfat thickness (mm)	17.68	17.91	17.84	18.01	18.01	18.05	0.15	0.0739	0.634
Lean (%)	52.06	52.60	52.24	52.74	52.75	52.88	0.29	0.045	0.835

Values represent the means of eight pens with four pigs per pen.

SEM, standard error of means.

1) CON, lysine:threonine = 1:0.65; Thr1, lysine:threonine = 1:0.66; Thr2, lysine:threonine = 1:0.67; Thr3, lysine:threonine = 1:0.68; Thr4, lysin:threonine = 1: 0.69; Thr5, lysine:threonine = 1:0.70.

**Table 5:** Effect of varying dietary Lys:Thr ratios on carcass trait and meat quality in finishing pigs

Items	Level of Thr (% of Lys requirements)						SEM	p-value	

	65	66	67	68	69	70			
	
	CON^1)^	Thr1^1)^	Thr2^1)^	Thr3^1)^	Thr4^1)^	Thr5^1)^		Linear	Quadratic
Carcass weight (kg)	90.41	91.09	90.97	91.03	90.50	91.59	0.92	0.583	0.961
Backfat thickness (mm)	18.56	19.41	19.19	19.25	19.13	19.66	0.65	0.387	0.841
pH	5.36	5.4	5.42	5.45	5.33	5.35	0.016	0.129	0.002
Water holding capacity (%)	38.87	45.56	39.2	43.73	37.57	46.05	1.532	0.206	0.49
Cooking loss (%)	30.63	31.22	30.8	32.35	30.81	32.81	0.455	0.005	0.54
Longissimus muscle area (cm^2^)	61.37	61.05	60.57	61.86	61.13	60.72	0.259	0.437	0.546
Drip loss (%)									
Day 1	4.36	4.67	4.46	4.6	4.29	4.96	0.285	0.401	0.60
Day 3	11.23	11.72	11.58	11.59	11.19	11.86	0.275	0.499	0.97
Day 5	15.80	16.62	15.54	15.99	16.19	16.84	0.314	0.101	0.15
Day 7	21.06	21.42	20.9	20.83	21.43	22.25	0.308	0.026	0.02
Meat color									
L*	47.14	46.97	49.99	48.13	47.57	47.98	0.677	0.468	0.07
a*	15.77	14.81	14.33	15.8	15.51	14.95	0.235	0.771	0.27
b*	4.33	4.35	4.24	4.51	4.38	4.15	0.115	0.584	0.21
Sensory evaluation									
Color	3.24	3.23	3.3	3.3	3.29	3.23	0.076	0.846	0.40
Firmness	3.20	3.3	3.36	3.25	3.21	3.3	0.089	0.496	0.59
Marbling	3.24	3.38	3.19	3.33	3.25	3.39	0.083	0.857	0.54

Values represent the means of randomly selected 10 pigs per treatment.

SEM, sStandard error of means.

1) CON, lysine:threonine = 1:0.65; Thr1, lysine:threonine = 1:0.66; Thr2, lysine:threonine = 1:0.67; Thr3, lysine:threonine = 1:0.68; Thr4, lysine:threonine = 1:0.69; Thr5, lysine:threonine = 1:0.70.
